# Gut Dysbiosis in Selected Gynecological Diseases Associated with Female Infertility: A Scoping Review

**DOI:** 10.3390/jcm15176705

**Published:** 2026-08-29

**Authors:** Łukasz Nowakowski, Katarzyna Warchoł, Agnieszka Kuźniar, Barbara Zimoń, Michał M. Skoczylas, Paweł Marzec, Grzegorz Krawczyk, Dorota Jegorow, Robert Pankiewicz, Andrew Z. Warchoł, Maciej Banach, Kinga Kuranty, Piotr Olcha

**Affiliations:** 1 Department of Gynaecology and Gynaecological Endocrinology, Medical University of Lublin, 20-049 Lublin, Poland; 2 Department of Genomics and Genetics, Faculty of Medicine, The John Paul II Catholic University of Lublin, 20-708 Lublin, Poland; 3 Institute of Medical Sciences, Faculty of Medicine, The John Paul II Catholic University of Lublin, 20-708 Lublin, Poland; 4 Institute of Economics and Finance, The John Paul II Catholic University of Lublin, 20-950 Lublin, Poland; 5 Independent Researcher, 20-340 Lublin, Poland; 6 Department of Preventive Cardiology and Lipidology, Medical University of Lodz, 93-338 Lodz, Poland

**Keywords:** endometriosis, infertility, gut microbiota, dysbiosis, inflammation, microbiome biomarkers, gut–ovary axis, mycobiota, probiotics, reproductive medicine

## Abstract

**Background/Objectives**: Female infertility represents a significant public health issue. Available evidence supports the hypothesis that the gut microbiota may play an essential role in women’s reproductive health and may serve as a diagnostic or prognostic biomarker in specific gynecological disorders. A substantial part of current research concerns disturbed communication between the hypothalamic–pituitary–ovarian axis and the gut microbiota, providing the basis for analyzing this phenomenon as the gut–ovary axis or the gut–vagina–ovary axis. The primary aim of this scoping review was to map the available evidence on the relationship between gut microbiota composition and female infertility, with particular emphasis on polycystic ovary syndrome (PCOS, currently polyendocrine metabolic ovarian syndrome, PMOS) endometriosis, and uterine fibroids. **Methods**: The review was conducted in accordance with the PRISMA Extension for Scoping Reviews (PRISMA-ScR). PubMed, Scopus, and Google Scholar were searched using terms related to gut microbiota, female infertility, PCOS, endometriosis, and uterine leiomyomas. Peer-reviewed publications in English published between 2015 and 2025 were considered. The included studies were descriptively synthesized to identify recurring microbiota patterns and research gaps. **Results**: The reviewed evidence indicates that gut dysbiosis may be associated with selected gynecological disorders affecting fertility, including PCOS, endometriosis, and uterine fibroids. The gut microbiome may have potential value as a biomarker supporting diagnosis, treatment selection, and prognosis. **Conclusions**: The gut microbiome represents a promising but still insufficiently validated area in the management of gynecological diseases associated with female infertility. Further high-quality clinical studies are needed to verify the effectiveness of microbiome-based therapies and to develop evidence-based guidelines for managing infertility associated with gut dysbiosis.

## 1. Introduction

### 1.1. Female Infertility and Its Causes

Female infertility affects approximately 186 million people in the world [1]. Anatomical–mechanical causes, such as tubal blockage, pelvic adhesions, and others, constitute over 30% and, together with endometriosis (with a varied clinical picture), an additional 15%. Ovulatory disorders, including polycystic ovary syndrome (PCOS), cause about a quarter of all fertility problems. Hormonal disorders are less common, the most common of which is hyperprolactinemia [1,2,3,4]. Tumors of the uterus, adnexa, and their surroundings can cause such problems as destructive lesions of the reproductive organs and mechanical obstructions. Among them, myomas are the most common benign tumors of the genital organs in women [5,6]. It is therefore clear that the structural and functional causes of infertility are intertwined in nature, and it is difficult to classify them based on disjoint sets. It is even more difficult to present the causes of infertility, which are often complex and not fully understood. An example can be endocrinopathies involving many hormones (e.g., prolactin, thyroid hormones, steroid hormones, and anti-Müllerian hormone) that have various pathogenesis, including genetic basis and environmental influences (e.g., endocrine-disrupting chemicals such as phthalates, cannabinoids, and alcohol consumed in an excessive way). Knowledge about the causes of diseases and failures in planning a pregnancy allows the development of appropriate treatment methods, for example inositol, biguanide antihyperglycemics, or glucagon-like peptide-1 for women with polycystic ovary syndrome [7,8,9,10,11,12,13,14]. A big challenge is idiopathic infertility; nevertheless, there is increasing information about this medical condition. The extensive but still insufficient knowledge about female infertility and its negative psychosocial effects indicate the necessity of holistic management of this health problem [15,16,17,18,19].

A significant part of the causes of female infertility are disorders caused by pathogenic microorganisms, including those leading to the development of pelvic inflammatory disease (especially Chlamydia trachomatis) and bacterial vaginosis [20,21].

### 1.2. Gut Microbiota and Female Reproductive Health

For many years, research results on microorganisms beneficial to human health have been reported repeatedly, but research efforts have provided only preliminary insights into the highly complex phenomena that include the chemical and physical interactions between individual microorganisms and between the microbiota and the human host under diverse physiological and pathological conditions, including distant body compartments [22,23,24,25,26,27,28,29,30,31,32,33,34]. It has been discovered, among others, that bacteria are capable of individual and collective movement and that gut microorganisms produce short-chain fatty acids (SCFAs), group B vitamins, and other bioactive metabolites, e.g., exopolysaccharides (EPS), which are known as postbiotics having a beneficial effect on human health, including antioxidant activity and, as in the case of EPS, inhibiting cholesterol absorption [35,36,37,38]. It is also known that chronic inflammation, both local and general, has an adverse effect on fertility, and microorganisms in the digestive and reproductive tracts are largely involved in this effect [39,40,41]. The primary focus of this review is the gut microbiota. However, evidence concerning the vaginal and endometrial microbiota is also included when relevant to interactions between the gut and the female reproductive tract. A substantial body of current research focuses on the disturbed communication between the hypothalamic–pituitary–ovarian axis and the gut microbiota, forming the basis for the concepts of the gut–ovary axis and the gut–vagina–ovary axis.

New discoveries and current knowledge suggest ways to diagnose conditions that already exist or are known to precede problems with conception. Broad-sense diagnostics in this area, based on metabolic testing, including hormone levels, inflammation, and body imaging of primarily the reproductive organs and pituitary gland, should be expanded to include newly discovered aspects of the whole-body microbiome, particularly in the reproductive tract and gastrointestinal tract, as indicated by the conclusions of this literature review [42,43,44,45,46,47,48,49].

The gut microbiota (both bacterial and fungal) remains in eubiosis, a balance between commensals (health-supporting bacteria and fungi), opportunists, and host management systems in physiological conditions. Intestinal dysbiosis or imbalance of microorganisms is a reduction in microbial diversity or an abnormal balance between commensal, opportunistic, and pathogenic microorganisms [50,51]. It can affect both bacteria and fungi (so-called mycological and bacterial dysbiosis). It is important to note that, although bacteria dominate the gut microbiome, fungi also play a significant role [50].

Although polycystic ovary syndrome (PCOS), endometriosis, and uterine fibroids differ in their clinical presentation and pathophysiology, they are discussed together because they are common causes of female infertility and have each been associated with alterations in the gut microbiota.

### 1.3. Clinical Context of the Selected Gynecological Conditions

Endometriosis, polycystic ovary syndrome/polyendocrine metabolic ovarian syndrome (PCOS/PMOS), and uterine fibroids are common gynecological conditions that may adversely affect female fertility, although the mechanisms involved are different [52,53,54,55,56,57,58,59,60,61,62,63,64,65,66]. Endometriosis is a chronic estrogen-dependent inflammatory disease associated with infertility. Its clinical course has also been studied in relation to inflammation, diet, and changes in the gut and reproductive tract microbiota [53,54,55,56,57,63]. PCOS/PMOS is a heterogeneous endocrine disorder associated with ovulatory, hormonal, and metabolic disturbances. Current literature also addresses nutritional, microbiome, and broader clinical aspects of the syndrome [52,58,59,60]. Uterine fibroids are benign hormone-dependent tumors of the uterus and may affect fertility depending on their clinical characteristics. Nutritional and microbiome-related factors have also been discussed, although microbiome evidence in fibroids remains limited [61,62]. These conditions represent distinct clinical entities and are therefore discussed separately in this review. Earlier longitudinal evidence also demonstrated that the vaginal microbiome is a dynamic ecosystem whose composition may vary over time, including across the menstrual cycle [66]. Vaginal and endometrial microbiota represent distinct microbial ecosystems and are mentioned only as contextual elements relevant to female reproductive health, whereas the primary evidence synthesis focuses on the gut microbiome [67].

### 1.4. Aim of the Review

This scoping review aims to map the available evidence regarding the gut microbiome profile in common gynecological conditions that impair female fertility, including polycystic ovary syndrome (PCOS), endometriosis, and uterine fibroids (leiomyomas). The review also summarizes the available evidence regarding the potential role of the gut microbiome as a biomarker for diagnosis, treatment, and prognosis in these conditions and identifies current research gaps. The review was guided by the following research question: What is known from the current literature about the relationship between gut microbiota composition and female infertility in women with polycystic ovary syndrome, endometriosis, and uterine fibroids?

## 2. Materials and Methods

### 2.1. Search Strategy and Information Sources

This scoping review was conducted in accordance with the PRISMA Extension for Scoping Reviews (PRISMA-ScR) guidelines. A completed PRISMA-ScR checklist is provided as Supplementary Materials, and the study selection process is presented in the PRISMA-ScR flow diagram. The review was not registered, and no protocol was prepared. The primary objective of this review was to map the available evidence regarding the relationship between the gut microbiota composition and female infertility, with a specific focus on three gynecological disorders: polycystic ovary syndrome (PCOS), endometriosis, and uterine fibroids/leiomyomas. The search strategy was based on a combination of relevant keywords and, in the case of PubMed, Medical Subject Headings (MeSH) terms. The following Boolean expression was used and adapted to each database’s syntax: “gut microbiota” AND “female infertility” AND (“polycystic ovary syndrome” OR PCOS OR endometriosis OR “uterine leiomyomas”). Due to the recent change in terminology, it should be noted that the literature search was conducted using the term Polycystic Ovary Syndrome (PCOS), as this was the accepted nomenclature at the time when the vast majority of the included studies were published and the primary indexing term used in bibliographic databases. In May 2026, following an international consensus, the condition was officially renamed Polyendocrine Metabolic Ovarian Syndrome (PMOS). Therefore, throughout this review, we use the designation PCOS/PMOS to ensure consistency with both the terminology used in the published literature and the current international nomenclature.

In PubMed, both keywords and MeSH terms were applied. In Scopus and Google Scholar, which do not support MeSH, the search was based exclusively on keywords. The complete electronic search strategy for PubMed is provided in the Supplementary Materials. Search strategies for Scopus and Google Scholar were adapted to the syntax of each database. The literature search was last conducted on 16 November 2025. In addition, the reference lists of eligible studies were screened to identify further relevant publications. Gray literature was not included in the search strategy.

### 2.2. Inclusion Criteria

The inclusion criteria were restricted to peer-reviewed publications published in English between 2015 and 2025, with full-text availability for analysis. English-language publications were selected to ensure consistent interpretation of the evidence and because English is the predominant language of microbiome research. A limited number of non-English sources were retained for contextual or methodological purposes and were not treated as direct disease-specific evidence. This time frame was selected to reflect substantial advances in microbiome analytical methods and the increasing standardization of clinical study designs, which together have enhanced the comparability and robustness of findings. Three publications published before 2015 were retained solely as foundational methodological or reference sources for Faith’s phylogenetic diversity, the Shannon diversity index, and the characteristics of the healthy human microbiome, respectively. Publications addressing the relationship between the gut microbiota and PCOS, endometriosis, uterine fibroids, or female infertility were considered eligible.

### 2.3. Exclusion Criteria

Case reports, conference abstracts without full texts, and animal or in vitro studies without direct relevance to the review question were excluded.

### 2.4. Data Charting

The following data were charted from each included study: author, year of publication, country, study design, study population, gynecological condition, microbiome assessment method, reported microbiome-derived indicators, principal findings, and methodological characteristics. No formal critical appraisal or risk-of-bias assessment was performed because the objective of this scoping review was to map the available evidence rather than to evaluate the methodological quality of individual studies.

### 2.5. Study Selection Process

**Figure 1:**
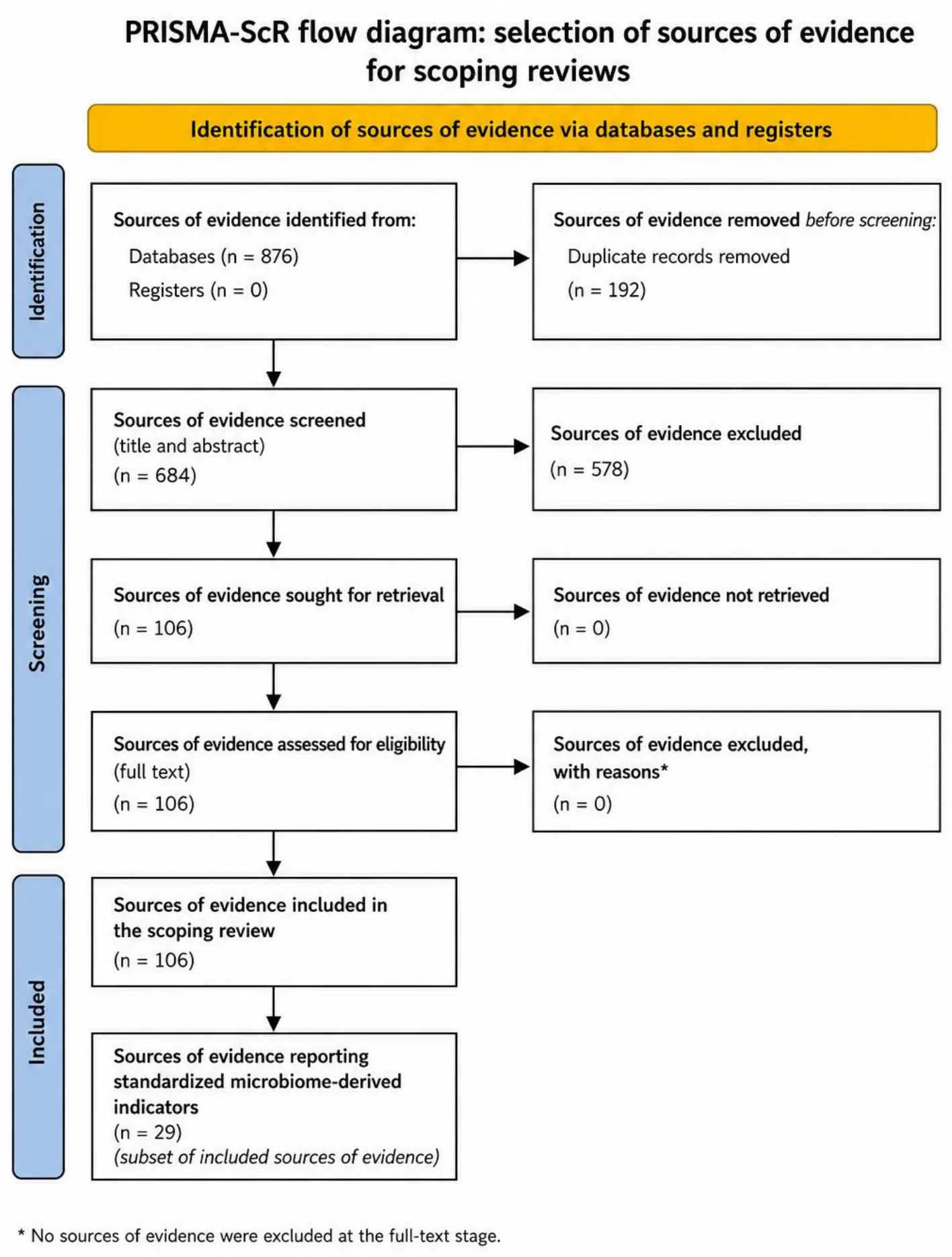
Flow diagram illustrating the study selection process for the scoping review. A total of 106 sources of evidence met the eligibility criteria and were included. Of these, 29 reported standardized microbiome-derived indicators. Adapted from the PRISMA 2020 flow diagram template, licensed under CC BY 4.0.

All retrieved publications were imported into the Mendeley Reference Manager (Elsevier, Amsterdam, The Netherlands) for manual duplication removal. The screening and study selection were performed independently by two reviewers, and any disagreements were resolved through discussion or adjudication by a third, senior author. A total of 876 records were identified, of which 192 duplicates were removed in the preliminary stage. The remaining 684 unique records were screened by title and abstract through independent dual-reviewer evaluation, following predefined inclusion and exclusion criteria. The full texts of 106 articles were assessed for eligibility, and all 106 met the eligibility criteria and were included in this scoping review; no articles were excluded at the full-text stage. Of these, 29 sources of evidence reported standardized microbiome-derived indicators and were descriptively summarized to facilitate comparison across studies (Figure 1).

### 2.6. Data Synthesis and Analysis

**Figure 2:**
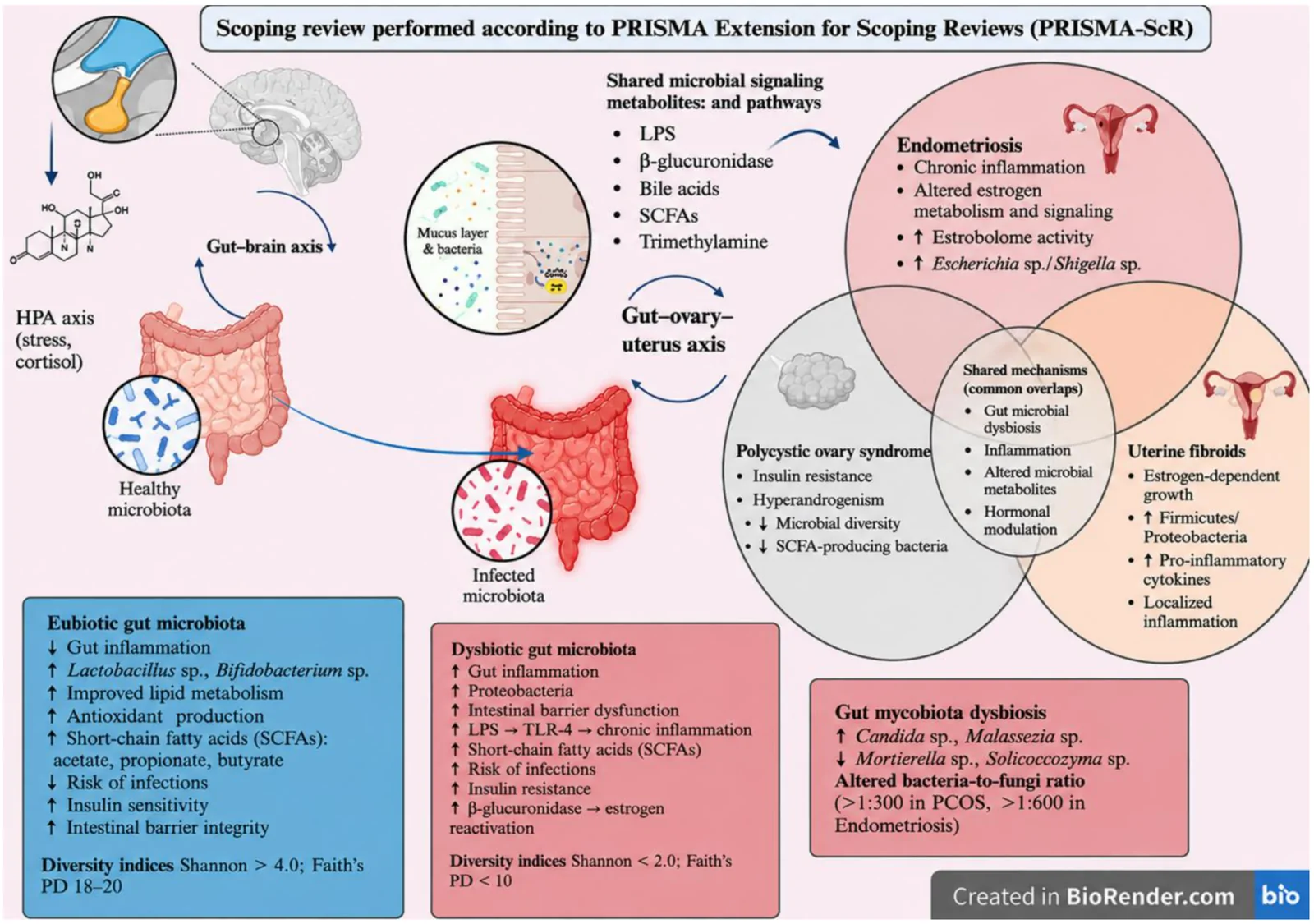
Conceptual overview of the proposed interactions between gut dysbiosis, microbiota-derived metabolites, the gut–brain–ovary axis, and selected gynecological diseases associated with female infertility. The figure summarizes the evidence identified in this scoping review and does not imply causal relationships. Abbreviations: PRISMA-ScR, PRISMA Extension for Scoping Reviews; HPA, hypothalamic–pituitary–adrenal; LPS, lipopolysaccharide; SCFAs, short-chain fatty acids; PD, phylogenetic diversity; PCOS, polycystic ovary syndrome. Symbols: ↑, increased; ↓, decreased. Arrows indicate proposed interactions or signaling pathways between the depicted components and do not imply causality. Created in BioRender. Warchoł, K. (2026) https://BioRender.com/h81dzc9 (accessed on 8 July 2026).

Among the included sources of evidence, 29 reported standardized microbiome-derived indicators that enabled descriptive comparison across studies. Alpha diversity based on Faith’s phylogenetic diversity (PD), the Firmicutes/Bacteroidota ratio, and Proteobacteria abundance were assessed across the included studies. Taxon-specific analyses included *Akkermansia* spp. and *Bifidobacterium* spp. Fungal-related indices, including *Candida* spp. abundance and fungal–bacterial interactions, and SCFA-related outcomes, including acetate:propionate:butyrate ratios, were also evaluated. Owing to substantial heterogeneity in study design, microbiome assessment methods, and reported outcome measures, no meta-analysis was performed. Instead, the findings were summarized descriptively and narratively and, where appropriate, presented in tables and figures to facilitate comparison across the included studies. The Discussion integrates findings from the included sources of evidence with additional literature used to provide clinical and methodological context (Figure 2).

## 3. Results

### 3.1. Overview of the Included Evidence

The results presented below summarize findings from the 106 sources of evidence included in this scoping review. Of these, 29 sources reported standardized microbiome-derived indicators, including α-diversity indices, phylogenetic diversity, taxonomic abundances, and SCFA-related markers, which were descriptively summarized to facilitate comparison across PCOS, endometriosis, and uterine fibroids.

### 3.2. Microbiome Findings in Endometriosis

#### 3.2.1. Gut Microbiome

The increased presence of Actinobacteria (13% vs. 4%) along with an increase in the proportion of Acidobacteria and other types (Cyanobacteria, Saccharibacteria, Fusobacteria) in patients with endometriosis was reported in the intestinal microbiota [68]. An elevated Firmicutes to Bacteroidetes ratio (3.55 vs. 1.99) was also observed in women with endometriosis [68,69,70]. The reduction in microbiota diversity in patients with endometriosis was reflected by lower Shannon and Simpson indices [70,71]. The lack of a significant change in the Chao Index may indicate that the main differences occur in evenness and dominance rather than in species richness [72].

#### 3.2.2. Gut Mycobiome

Regarding the intestinal mycobiota, Ascomycota and Basidiomycota dominate, as in healthy individuals, and significant differences in endometriosis more often concern the architecture of fungal–bacterial interactions than the percentage shares themselves [73,74]. The comparative microbiota profiles are summarized in Table 1.

### 3.3. Microbiome Findings in PCOS/PMOS

An alpha diversity analysis was conducted to assess the richness, diversity, and evenness of gut microbial communities, using such indicators as the number of observed taxonomic units (OTUs), Chao1, Shannon, Simpson, and Pielou_e. Numerous microbiological examinations of the feces of patients with polycystic ovary syndrome (PCOS) showed a significantly lower number of observed OTUs compared to control subjects. In addition, the PCOS/PMOS patients had a significantly lower Chao1 index, indicating reduced microbial richness in stool samples. In addition, significantly reduced Shannon and Simpson diversity indices were observed in the PCOS/PMOS subjects compared to healthy controls. The reduced uniformity of taxon distribution was also confirmed by the Pielou_e index [75,76,77,78].

#### 3.3.1. Gut Microbiome

The analysis of the fecal bacteriomes of the study participants revealed that the composition of the intestinal microbiota was dominated by four main types (phyla): Firmicutes, Bacteroidetes, Proteobacteria, and Actinobacteria. Together, these four taxonomic groups accounted for more than 90% of the total abundance of bacteria present in the samples, demonstrating their crucial role in the intestinal ecosystem in both healthy and PCOS/PMOS women [75,76,77,78]. At the genus level, the most prevalent bacteria were as follows:

*Bacteroides*—typical of the gut, associated with fat and carbohydrate metabolism;

*Prevotella_9*—often found in the microbiota of people who consume a lot of fiber;

*Faecalibacterium*—known for its anti-inflammatory properties and butyrate production;

*Roseburia*—producers of butyrate supporting the health of the intestinal epithelium.

The presence of these genera reflects the typical composition of a healthy intestinal microflora, although their proportions differed significantly between the study groups, especially in the context of PCOS/PMOS and BMI [79,80,81].

#### 3.3.2. Gut Mycobiome

Women with polycystic ovary syndrome were reported to have an elevated abundance of fungi in the mycobiome belonging to the genera *Candida* and *Malassezia* as well as less common taxa, such as *Kazachstania* sp., *Microascus* sp., *Coniochaeta* sp., *Xepicula* sp., *Paraphoma* sp., *Pyrenochaetopsis* sp., *Cephaliophora* sp., *Epicoccum* sp., and *Sclerophora* sp. [79].

The composition of the intestinal mycobiota differed significantly between women with PCOS/PMOS and healthy women [79,80,81].

- Ascomycota—the predominant fungal type in all groups: healthy and PCOS, with a proportion ranging from ~50% in the mycobiome of healthy women to ~80% in the mycobiome of women with PCOS. This type includes many common fungi, e.g., *Candida* sp., *Aspergillus* sp., and *Kazachstania* sp.
- Basidiomycota—the second most common type, more prominent in healthy groups. The proportion ranges from ~10% to ~20%.
- Mortierellomycota—noticeably increased only in healthy women (~15–20%), suggesting a possible protective effect of this group of fungi in women.
- *Candida* sp.—a genus often associated with metabolic disorders and inflammation. Its abundance was significantly higher in women with PCOS.
- *Mortierella* sp.—much more abundant in the mycobiome of healthy women (~20–25%); almost absent in groups with PCOS.
- *Solicoccozyma* sp.—marked abundance in the mycobiome of healthy women (~10%); less well represented in other groups.
- *Malassezia* sp.—a higher proportion was reported in PCOS/PMOS groups (~10%).

### 3.4. Gut Microbiome Findings in Uterine Fibroids

Evidence regarding gut microbiome alterations in women with uterine fibroids remains limited. An elevated Firmicutes/Bacteroidota ratio and increased Firmicutes abundance have been reported in women with uterine fibroids [61]. Descriptive comparisons also suggested mild and inconsistent reductions in microbial diversity, together with alterations in taxonomic composition and selected microbiome-derived indicators. Overall, these findings were less consistent than those reported for PCOS/PMOS and endometriosis.

### 3.5. Overall Patterns Across Conditions

Overall, the included sources of evidence suggest that gut microbiota alterations are associated with PCOS, endometriosis, and uterine fibroids, although findings vary across studies. Changes were reported in microbial diversity, taxonomic composition, and SCFA-related markers, with more consistent findings for PCOS/PMOS and endometriosis than for uterine fibroids.

## 4. Discussion

### 4.1. Principal Findings

This scoping review identified gut microbiota alterations across PCOS/PMOS, endometriosis, and uterine fibroids, with more consistent evidence for PCOS/PMOS and endometriosis. Gut microbiota represent distinct microbial ecosystems and are considered only as complementary contextual evidence; they are not part of the primary gut microbiome synthesis.

### 4.2. Interpretation of Disease-Specific Microbiome Patterns

Although the primary focus of this review is the gut microbiome, hormonal influences on gut microbial communities provide relevant contextual information. Previous studies have reported associations between hormonal treatment or menstrual-cycle phases and changes in *Lactobacillus* sp., *Atopobium* sp., *Prevotella* sp., and *Sneathia* sp. abundance [64,65,66,67]. These findings concern distinct reproductive-tract microbial niches and are not interpreted as direct evidence of gut microbiome alterations.

Hormonal changes have a significant impact on the composition of the endometrial microbiome. Exogenous progestogens, which are widely used in the treatment of endometriosis, significantly alter the endometrial microflora, e.g., reducing the diversity of *Lactobacillus* sp. bacteria. In addition, they increase the diversity of the gut microbiome, and the abundance of such bacteria as *Atopobium* sp. and *Prevotella* sp. increases after hormone treatment, while the percentage of *Lactobacillus* decreases slightly [64,65]. It is worth noting that naturally occurring hormonal fluctuations during the menstrual cycle correlate with the instability of the microbial population [66]. Significant changes also occur in the endometrial microbiome. Increased numbers of *Prevotella* sp. and *Sneathia* sp. bacteria may be characteristic of the proliferative and secretory phases, respectively [67].

#### 4.2.1. Endometriosis

These findings suggest that functional couplings (e.g., the influence of fungal metabolites on bacteria and vice versa) may be more important for the pathophysiology of endometriosis than simple quantitative changes at the phylum level [73,74].

#### 4.2.2. PCOS/PMOS

Reduced gut microbiota diversity and richness indicates its reduced diversity and richness in women with PCOS, which may be associated with implications for metabolic regulation, immune response, and the gut–ovarian axis. Abnormalities in the microbiota composition and diversity may be associated with the pathophysiology of PCOS/PMOS, affecting insulin resistance, inflammation, and hormonal balance, among other factors [75,76,77,78].

Greater importance is placed on the functional dimension, where a “healthy microbiota” is defined not so much by specific taxa but by their metabolic activity, ability to interact with the host, and impact on intestinal barrier integrity, the extended system, and systemic homeostasis. Therefore, assessing the functions performed by microorganisms, such as short-chain fatty acid (SCFA) production and modulation of the basal state, or their identification and use, is crucial, rather than solely defining these functions. Furthermore, the composition of the gut microbiota is influenced by numerous confounding factors, such as genetic predisposition, diet, environment, age, use of antibiotics (if necessary), and psychosocial factors, including stress. These determinants, in time and space, further complicate the attempt to universally define a healthy gut microbiota. In summary, current research models advocate a shift from the existing healthy microbiota as the sole taxonomic criteria and return to integrated functional approaches, physiological microbiota–host interactions, and the functional nature of the microbiome [61,62,63,82].

### 4.3. Microbiota and Mycobiota as a Biomarker

The human microbiota, particularly the intestinal microbiota, is involved in maintaining the body’s immunological, metabolic, and endocrine homeostasis. Disturbances in its composition (dysbiosis) have been associated with numerous conditions, such as irritable bowel syndrome (IBS), obesity and metabolic syndrome, endometriosis and infertility [83,84,85], bacterial vaginosis and preterm birth [86,87], and gastrointestinal and reproductive cancers [88,89]. The human gastrointestinal tract provides a habitat for a complex community of microorganisms—bacteria, archaea, viruses, yeasts, and fungi—which constitute the so-called gut microbiota. Increasingly, the microbiota and its collective material, termed the “second genome”, are recognized as key components of the host’s homeostasis [90]. The gut microbiome participates in the regulation of numerous pathogenic factors, including metabolism, systemic development, and mucosal barrier function. Furthermore, gut microorganisms produce short-chain fatty acids (SCFAs), B and K vitamins, and other bioactive metabolites, which are additional to the host in the absence of microbiota [91,92]. These substances not only provide energy but also influence the integrity of the intestinal epithelium, local and systemic immune responses, and the gut–brain axis [93,94,95,96]. Alterations in microbiota composition and function have been associated with inflammatory and metabolic disorders [92,97,98]. Because the microbiota dynamically responds to environmental and host-related factors, its analysis, particularly using diversity indices, has been investigated as a potential source of non-invasive biomarkers of pathological changes.

We suggest that selected biodiversity indicators may serve as potential tools for assessing the microbiota in gynecological conditions affecting fertility. Evidence from PCOS/PMOS supports their relevance, and preliminary findings indicate similar patterns in endometriosis. In contrast, evidence regarding uterine fibroids remains scarce, and the available studies are insufficient to draw firm conclusions. Therefore, while these observations point to a possible broader diagnostic potential, particularly for PCOS/PMOS and endometriosis, the applicability of biodiversity indicators to uterine fibroids requires further validation in well-designed, large-scale studies [69,70,71,76,77,79].

Faith’s PD is a measure of phylogenetic diversity that takes into account not only the number of species (richness) but also their evolutionary distances [99]. It counts the length of all branches of the phylogenetic tree, encompassing the observed taxa. Faith’s PD considers the evolutionary depth of organisms, making it a sensitive marker of the functional complexity of the microbiota. A higher Faith’s PD value indicates that the microbiota contains more evolutionarily diverse organisms, which translates into greater functional potential, for example, in terms of metabolism, immune response, and protection against pathogens. Table 2 presents a potential interpretation of Faith’s PD applied to gynecological diseases. A high Faith’s PD indicates greater evolutionary diversity and functional potential of the microbial community, which is associated with resistance to disruption, a stronger mucosal barrier, and a beneficial effect on hormone metabolism and inflammation. Low values are characteristic of dysbiosis, inflammation, and pathobiont dominance [100,101].

The presented ranges are illustrative, context-dependent, and derived from heterogeneous studies; they should not be interpreted as validated clinical cut-off values.

In women with PCOS/PMOS, a significantly lower Faith’s PD (*p* = 0.02) suggests that the microbiota is not only less species-rich but also less functionally diverse. This may limit its ability to produce beneficial metabolites (e.g., SCFAs), maintain intestinal barrier integrity, and modulate the immune and hormonal systems. Lower phylogenetic diversity is associated with greater susceptibility to dysbiosis and metabolic disorders, which is part of the pathogenesis of PCOS/PMOS (e.g., insulin resistance, chronic inflammation) [76,77,82,84,100,101,102,103,104,105].

The Shannon–Wiener index (denoted H or H’) is a measure of biodiversity in a sample. In the context of the microbiome, it describes taxonomic richness (the number of species) and species evenness (how evenly distributed the abundances of different taxa are). There are scientific reports that the Shannon Index can be used in clinical studies as an indicator of the state of the microbiological ecosystem, e.g., in the analysis of intestinal, vaginal, skin, or oral microbiota, studies on dysbiosis in chronic diseases (obesity, diabetes, depression, Parkinson’s disease), assessments of the impact of antibiotics, diet, probiotics, and hormones on the diversity of microorganisms, and prediction of the response to immunological or hormonal treatment [103] (Table 3).

A Shannon–Wiener index value below 2.0 results from dysbiosis, i.e., a microbiological imbalance that can occur with increased risk of developing a condition, hormonal influences, and infertility. These recommendations include recommendations for restoring the microbiota, such as the use of probiotics, the use of gut microbiota therapy (FMT), targeted antimicrobial or immunomodulatory therapies, and hormonal support.

These are indicative, context-dependent ranges derived from synthesized microbiota studies and are not intended as universal clinical cut-off values.

An index range of 2.0–4.0 has been described in some studies as an intermediate or transitional range between lower and higher microbial diversity. At this stage, preventative measures, such as stress reduction, lifestyle changes, and dietary modifications (e.g., increasing dietary fiber and prebiotics), are crucial, as they can support the restoration of microbial homeostasis. Shannon–Wiener index values above 4.0 have been reported in studies describing microbiota with relatively high diversity and evenness. This state promotes the maintenance of metabolic, immunological, and hormonal homeostasis, which is crucial for overall health. In this case, supportive measures are recommended, particularly consuming a diet rich in fiber and prebiotics and avoiding factors that disrupt microbiota (e.g., antibiotics, stress, highly processed foods). The Shannon–Wiener index may be useful for characterizing microbiota diversity; however, the ranges presented in Table 3 should be interpreted as context-dependent rather than as validated diagnostic thresholds (Table 3) [63,76,79,80,82,101,102,107,108,109,110].

Reduced taxonomic diversity and altered bacterial distribution may be associated with changes in sex hormone metabolism and inflammatory responses. However, the clinical utility of the H’ index as a diagnostic biomarker or therapeutic target remains uncertain.

The F/B ratio (Firmicutes to Bacteroidota ratio) is an indicator describing the relative grouping of two characteristic features of the human gut microbiota. Firmicutes includes other *Lactobacillus* sp., *Clostridium* sp., *Faecalibacterium* sp., and *Ruminococcus* sp. They are important in fiber breakdown, butyrate production, and ductal fermentation. Bacteroidota (formerly Bacteroidetes) includes some *Bacteroides* sp. and *Prevotella* sp., among others. They are responsible for the degradation of plant polysaccharides, bile acid metabolism, and immune protection. The optimal value in a healthy woman is usually F/B = 1.5–2.0. The increased F/B index, reflecting a relative increase in Firmicutes and a simultaneous decrease in Bacteroidota, is considered one of the main markers of intestinal dysbiosis and is associated with several adverse metabolic and hormonal consequences. In PCOS/PMOS, an increased F/B ratio correlates with increased hyperandrogenism, higher body mass index (BMI), insulin resistance, and higher fasting insulin levels. These observations are supported by studies conducted by Talwar et al. (2022) and Yurtdaş and Akdevelioğlu (2020), suggesting that intestinal dysbiosis may play a significant role in the pathogenesis of the metabolic phenotype of PCOS [104,105].

In uterine fibroids, the reported increase in Firmicutes abundance may be associated with increased estrogen circulation through increased enzyme activity in the gut microbiota, which may influence the development and progression of estrogen-dependent tumors [66]. The physiological consequences of an increased F/B ratio include increased fermentation of indigestible carbohydrates, leading to greater energy absorption and potential weight gain, reduced production of short-chain fatty acids (SCFAs), including butyrate, which plays a key role in maintaining intestinal barrier integrity and exhibits anti-inflammatory effects, increased intestinal permeability and the penetration of lipopolysaccharides (LPS) into the systemic circulation, resulting in low-threshold inflammation and metabolic endotoxemia, and increased enzymatic activity of Firmicutes microorganisms, including such enzymes as β-glucuronidase, which are involved in estrogen recirculation (enterohepatic circulation) and may be important in estrogen-dependent diseases such as uterine fibroids or endometriosis. Therefore, an elevated F/B ratio not only reflects disturbed microbiota homeostasis but may also actively participate in the pathogenesis of metabolic and gynecological diseases through immunological, hormonal, and metabolic mechanisms. To contextualize current evidence, we identified the most frequently reported microbiological parameters in recent literature and summarize them in Table 4. Indicators were selected based on reproducibility in high-quality studies published between 2019 and 2025, ensuring robustness and consistency of the available evidence [58,61,72,76,79,82,99,101,104,105,106,107,110,111,112,113,114,115,116,117,118,119,120,121].

Increased share of Proteobacteria in the microbiota profile. These bacteria, including the genera *Escherichia*, *Klebsiella*, *Enterobacter*, and *Salmonella*, constitute a diverse group of Gram-negative bacteria that occur in low proportions in the gut microbiota of women in physiological conditions (<2% relative share). It is important to emphasize the importance of a molecular mechanism here: the LPS–TLR4–inflammatory–endocrine axis. Gram-negative bacteria of the Proteobacteria phylum synthesize lipopolysaccharide (LPS), the main component of their outer membrane. LPS acts as a potent proinflammatory factor capable of activating the host immune system. Another aspect is the translocation of LPS and the crucial role of the intestines in this process. Under dysbiosis and impaired intestinal barrier integrity (so-called leaky gut), LPS can enter the bloodstream and induce a systemic inflammatory response. This aspect can trigger the LPS signaling pathway, and thus TLR4 and NF-κB. Once in circulation, LPS binds to Toll-like receptor 4 (TLR4) present on immune cells (monocytes, macrophages) and metabolic tissues. The activation of this pathway leads to recruitment of the MyD88 adaptor protein, activation of the NF-κB transcription factor, and increased expression of genes encoding proinflammatory cytokines: IL-6, IL-1β, and TNF-α.

The activation of the LPS-TLR4 axis has been associated with chronic low-grade inflammation and endocrine and metabolic disturbances observed in PCOS. Insulin resistance has been associated with proinflammatory cytokines, impaired insulin receptor (IR) signaling, and hyperinsulinemia. Another important aspect is hyperandrogenism. Insulin may stimulate ovarian theca cells and contribute to increased androgen production, including testosterone and androstenedione. Another important issue is menstrual cycle disruption, which has been associated with chronic inflammation and altered hormonal profiles that may affect follicular maturation and ovulation. Finally, a crucial aspect focuses on issues related to aromatase and estrogen. IL-6 and TNF-α have been shown to stimulate aromatase expression, disrupting estrogen balance.

### 4.4. Clinical Implications and Future Perspectives

The available evidence suggests that disturbances in the gut–brain–ovary axis may be associated with hormonal and metabolic dysregulation affecting female fertility. There is growing interest in the use of microbiota-directed interventions, particularly probiotics, prebiotics, synbiotics, dietary strategies, and FMT, as adjuncts or potential therapeutic options in the treatment of female infertility. However, current evidence is limited by the scarcity of well-designed randomized controlled trials and long-term observational studies that could confirm causal relationships and establish standardized intervention protocols. Available evidence suggests that alterations in the gut microbiota may contribute to female reproductive disorders and may have potential as diagnostic or prognostic biomarkers in selected gynecological diseases. Further high-quality clinical trials are warranted to verify the effectiveness of microbiota-based interventions and to develop evidence-based therapeutic guidelines for the management of infertility associated with gut dysbiosis.

## 5. Safety, Limitations, and Future Directions

### 5.1. Importance of Appropriate Selection of Research Design and Sample Size in Microbiota Research

This scoping review has several limitations. The search was limited to PubMed, Scopus, and Google Scholar and to English-language publications from 2015 to 2025, which may have resulted in relevant evidence being missed. No formal critical appraisal of the included studies was performed. In addition, the available evidence is affected by methodological limitations. Many microbiota studies are characterized by suboptimal study designs and insufficient sample sizes, which can significantly impact the reliability and generalizability of their results. The study design should be closely aligned with the research questions and hypotheses, as it determines the validity and interpretability of conclusions regarding the relationship between microbiota and health.

It should be added that cross-sectional studies are particularly useful in identifying correlations between the microbial community structure and clinical parameters, although they do not allow determining the direction of the relationship or causality. Case–control studies, on the other hand, facilitate identification of potential biomarkers of health or disease but are susceptible to biases related to the selection of comparison groups and confounding factors [52,63,82,123,124,125,126,127,128,129,130,131,132,133,134,135,136,137,138,139,140,141,142,143,144,145,146,147,148].

While longitudinal studies provide a valuable source of information on the dynamics of changes within the gut microbiota, allowing monitoring its composition over time and identifying the impact of environmental, dietary, pharmacological, or psychological factors on its stability and functionality, randomized controlled trials (RCTs) are considered the gold standard in interventional research, as they contribute to identification of cause-and-effect relationships between changes in the microbiome and clinical or health parameters [122,126].

### 5.2. Ability to Distinguish Disease-Specific Microbiota Patterns

Analyses of the gut microbiota often focus on a single disease entity, whereas comparative studies across multiple conditions can reveal common changes in the microbial community of the gastrointestinal tract. Damage to the mucosal layer covering the intestinal epithelium coupled with inflammation is observed in patients with endometriosis, polycystic ovary syndrome (PCOS), and uterine fibroids as well as in inflammatory bowel disease (IBD), celiac disease, HIV-associated enteropathy, acute diarrhea, cancer, and irritable bowel syndrome (IBS). Given these similarities, it can be assumed that specific microbial groups may exhibit similar trends in their abundance or decline across different disease entities. However, a full understanding of these relationships may require detailed biogeographic analyses along the entire gut, which will become possible with the development of safe and reliable methods for comprehensive sampling along the gastrointestinal tract [61,63,82,90,97,128,129,130].

### 5.3. Technical Variability and Lack of Standardization of Analytical Protocols in Gut Microbiota Studies

Biological and Analytical Consequences of Incorrect Sample Collection

The findings presented in Table 5 show an overgrowth of opportunistic aerobes (e.g., *Enterococcus* sp., *Escherichia* sp.) with a simultaneous decline in strictly anaerobic bacteria (*Faecalibacterium prausnitzii*, *Bacteroides fragilis*). Importantly, from the perspective of patient outcomes, the results may be biased in terms of alpha and beta diversity indices (Shannon, Faith’s PD), preventing comparisons between samples and studies. Another significant risk is the disruption of functional analyses (e.g., metagenomics, metatranscriptomics) and erroneous results for metabolic profiles (SCFA, estrogen metabolism). Data reproducibility is a problem for studies specifically related to PCOS/PMOS and endometriosis. These are unusual situations where the effects are subtle, and the differences between patients and control groups are susceptible to analytical artifacts (Table 5).

There are some reports in the literature regarding technical challenges in microbiota analysis. Vandeputte et al. (2017) demonstrated that differences in microbial load between samples may influence the interpretation of microbiota composition [131]. Bundgaard-Nielsen et al. (2018) found that different storage conditions may affect bacterial composition and diversity, although inter-individual differences were greater than those related to storage conditions [132]. On the other hand, Tunsakul et al. (2024) reported that aerobic collection and transport (<48 h) had no statistically significant effect on fecal microbiota, and no statistically significant differences were observed in quantitative microbiota analyses or alpha and beta diversity measurements [133]. The study also found that specific container-based collection and transport still allowed the differentiation of fecal microbiota in individuals with lipid metabolism disturbances, similar to anaerobic sampling and transport. A study conducted by Antosca et al. 2020 and Hoogendijk et al. 2024 demonstrated that the use of commercial stabilizers (OMNIgene-GUT) ensures a microbiota profile comparable to that determined after freezing the sample at −80 °C but may affect the efficiency of DNA extraction in some bacterial groups [134,135,136,137].

This table summarizes disease-specific methodological factors reported in studies of gut microbiome and mycobiome alterations in PCOS/PMOS endometriosis and uterine fibroids; however evidence for fungal alterations, particularly in uterine fibroids, is limited and should be interpreted with caution.

One of the main sources of discrepancies in the results of gut microbiota analyses is the lack of consensus on the selection of the variable region of the 16S rRNA gene to be amplified and sequenced. This gene contains nine variable regions (V1–V9), of which V1–V2, V3–V4, V4, and V6–V8 are most commonly analyzed. Each of these regions has a different ability to distinguish specific bacterial taxa, which affects the final taxonomic composition identified in the sample. For example, the V1–V2 regions may be better at detecting bacteria of the genus *Bifidobacterium*, while the V4 region offers broader phylogenetic coverage and better comparability between samples. However, different regions vary in length, contain different conserved and variable sequences, and thus lead to varying levels of taxonomic resolution: from the genus to family or phylum level. The choice of the amplified 16S rRNA region can influence the observed microbiome composition and taxonomic resolution, potentially resulting in underestimation, misclassification, or omission of certain bacterial taxa [139,140,141,142,143,144].

In response to this problem, the global Earth Microbiome Project (EMP), part of the broader Earth Human Microbiome (EHM) program, has proposed a standard approach to analyzing microbiomes on a planetary scale. The EMP has adopted the V4 region of the 16S rRNA gene as the standard for analyzing the microbiome of bacteria and archaea, based on its compromise properties: moderate length, broad taxonomic coverage, and a good signal-to-noise ratio. Standardization of this region has ensured comparability of results between studies, improved the quality of meta-analyses, and facilitated the creation of global microbiome databases.

For eukaryotic microorganisms such as fungi, the EHM and EMP recommend the use of ITS (Internal Transcribed Spacer) markers, especially the ITS1 or ITS2 region, which show high variability and allow identification of fungi at the species level. Again, the lack of a uniform approach to ITS region selection can lead to inconsistent results, especially in gut microbiota studies involving commensal or opportunistic fungi.

The implementation of recommendations from such projects as EMP/EHM and the use of the same marker regions (e.g., V4 for bacteria, ITS1/ITS2 for fungi) represents an important step toward standardizing microbiome research. For clinical diagnostics and translational research, this means greater consistency, the possibility to compare results over time and between centers, and a better basis for developing standardized indicators of dysbiosis.

Another problem may be the choice of sequencing platforms. Short reads (150–300 bp), high accuracy (>99.9%), and mature bioinformatics pipelines make amplicon sequencing of 16S rRNA (bacteria) and ITS (fungi) regions a standard in microbiota and mycobiota research, including gynecology [144,145,146,147,148,151,152]. Current guidelines include protocols specific to vaginal microbiome profiling and combining 16S and ITS markers in a single project [139,140,141]. High-throughput taxonomic profiling (e.g., CST, alpha/beta diversity); cohort comparisons (endometriosis, infertility, IVF vs. a healthy group); and identification of markers of dysbiosis associated with pregnancy complications may be clinically significant. An unfavorable feature is the lack of full strain resolution and information on functional genes (e.g., β-glucuronidase affecting estrogen metabolism). Results depend on primer selection, region length, and DNA extraction efficiency [142,143,147,152,153,154].

In infertility and endometriosis research, the choice of sequencing platform has a critical impact on the resolution and interpretability of microbial community analyses. Short-read platforms, such as Illumina MiSeq or NovaSeq, offer high sequencing accuracy and depth, enabling robust quantification of relative abundance and diversity indices (e.g., Shannon index, Faith’s phylogenetic diversity). However, the short read length may limit precise resolution at the species and strain level, which may impact phylogenetic analyses [140,141,142].

In contrast, long-read technologies, such as PacBio and Oxford Nanopore, provide longer read lengths, facilitating better assembly and strain-level resolution, although this comes with a higher number of sequencing errors per read. These platforms are particularly valuable for characterizing complex microbial communities where closely related taxa coexist.

In reproductive health research, accurate differentiation of *Lactobacillus* strains is critical, as different strains exhibit diverse immunomodulatory and metabolic properties that can influence inflammation, hormonal regulation, and therapeutic responses. Long-read sequencing therefore enables better identification of strain-specific functions, which is important for the development of strain-targeted probiotic interventions aimed at modulating the microbiota in patients [140,141,142].

### 5.4. Problems with Analysis of the Gut Mycobiome

Research on the role of the gut mycobiome in gynecological conditions, such as polycystic ovary syndrome (PCOS), endometriosis, and uterine fibroids, is gaining importance. A growing number of reports indicate that disturbances in the composition and balance of intestinal fungi, such as excessive *Candida* spp. overgrowth or reduced species diversity, may contribute to the development and progression of these conditions by modulating the immune system and hormone metabolism [153,154]. Although intestinal fungi constitute a minority in the microbiota relative to bacteria, they play an important role in intestinal homeostasis and modulation of the immune system and hormone metabolism. As with bacterial analysis, the quality and standardization of stool sample collection and storage are crucial, particularly due to the sensitivity of fungi to environmental factors and rapid changes in the population composition during inappropriate transport.

### 5.5. Lack of a Single Method for Assessing an “Unfavorable Gut Microbiota Profile”

In recent years, the risk of gut microbiota contamination in infection and disease has increased. Disturbances in the components and functions of the microbiome are associated with numerous disease entities, including metabolic, autoimmune, neuropsychiatric, and cancer symptoms. Despite the use of microbiome research, there are still no uniform, verified, and validated criteria for assessing an “unfavorable gut microbiota profile”. Currently available diagnostic methods differ in their assessments (e.g., 16S rRNA/ITS delivery vs. shotgun metagenomics), the range of taxa analyzed, result interpretation, and reference standards. In practice, this is hampered by results of tests conducted in different laboratories, limiting their diagnostic usefulness and constituting a barrier to consistent therapeutic recommendations. The lack of standardization affects not only the laboratory procedures themselves but also the metrics for “healthy” and “disturbed” microbiomes. No definitive thresholds or indicators have been established to objectively classify a microbiota profile as pathogenic or controlled independently. Consistent indicators of dysbiosis are also available, which could provide a taxonomic solution and potentially functional microorganisms. Introducing standardization in gut microbiota diagnostics requires multifaceted efforts: establishing a collection and storage protocol, validating analytical methods, establishing uniform bioinformatics frameworks, and developing standardized reference databases. Interdisciplinary collaboration is also necessary, encompassing microbiologists, bioinformaticians, clinicians, and institutions [155]. Understanding the use of microbiota is crucial for the development of microbiotic medicine and the implementation of effective therapeutic strategies based on microbiome modulation. Without standardization, using microbiota as a diagnostic and prognostic tool will merely be a solution for experimental applications [63,82,90,156].

The reviewed studies show substantial methodological heterogeneity, because different sequencing platforms, 16S/ITS regions, and bioinformatic pipelines introduce technical bias and hinder cross-study comparability. Variations in read processing, taxonomic classifiers, and reference databases further affect the detection of microbial signatures. Greater standardization of protocols, as recommended by initiatives such as the Earth Microbiome Project, is essential to improve reproducibility and support more robust integration of findings.

### 5.6. Endometriosis, PCOS/PMOS and Uterine Fibroids as a Source of Socio-Economic Costs

Beyond the potential clinical implications, these conditions also impose a substantial socio-economic burden, further highlighting the importance of early diagnosis and effective management. Costs related to chronic gynaecological conditions, including endometriosis, polycystic ovary syndrome and uterine fibroids, include direct, indirect and social costs. Direct costs include expenses for diagnostics, treatment and hospitalisation, while indirect costs are due to loss of productivity, sickness absence and reduced labour force participation of women. Social costs include reduced quality of life and mental and family burdens. Studies show that indirect costs often outweigh direct costs and remain insufficiently accounted for in systems analyses [155,157,158,159,160,161].

Multi-centre studies conducted in Europe, the United Kingdom and the United States found that the annual cost of treatment for one woman with endometriosis was €9579, most of which was indirect costs related to absenteeism (€6298). Endometriosis generates significant, often underestimated, societal costs due to diagnostic delays and the chronic nature of the disease, and its economic burden is comparable to or even higher than that of other chronic diseases, such as cardiovascular disease or diabetes [155,157,162].

PCOS/PMOS is a chronic condition with numerous metabolic and reproductive disorders, the economic cost of which in the US in 2020 was estimated at USD 7.9 billion, of which USD 3.9 billion was related to comorbidities [163,164].

Uterine fibroids are one of the main causes of gynaecological procedures, increasing the burden on the healthcare system, and perinatal complications further increase the cost of the disease [165,166].

Endometriosis, PCOS/PMOS, and uterine fibroids are examples of chronic gynecological diseases in which hormonal imbalance, chronic inflammation, and metabolic dysfunctions intertwine with significant social and economic consequences [155,157,167,168]. Clinical management of endometriosis requires an individualized approach [169]. In recent years, increased attention has been paid to the role of dysbiosis of the gut and reproductive tract microbiome as a potential factor linking these pathophysiological mechanisms [170,171]. Disruptions of the estrogen microbiome axis (estrobolome) may affect estrogen bioavailability, immune response, and the persistence of chronic inflammation, which is relevant to the development and progression of hormone-dependent diseases [170].

From a health economics perspective, these diseases generate substantial direct costs related to diagnostics, and pharmacological and surgical treatment, as well as indirect costs resulting from productivity loss and reduced quality of life among women of a working age [157,167]. Available data indicates that the greatest financial burden on a healthcare system occurs at the advanced stages of disease, when hospitalizations, repeated surgical interventions, and treatment of metabolic and reproductive complications are required [167]. In this context, microbiome modulation through diet, lifestyle, and interventions supporting hormonal immunological balance appears as a potential component of a strategy shifting the focus from reactive treatment to prevention, which in the long term may lead to a reduction in treatment costs and improvement in health outcomes (Table 6) [170].

## 6. Conclusions

This review highlights the growing evidence that gut dysbiosis is associated with the pathophysiology of endometriosis, polycystic ovary syndrome/polyendocrine metabolic ovarian syndrome (PCOS/PMOS), and uterine fibroids. Across these conditions, common microbial signatures—including reduced alpha diversity, an increased Firmicutes/Bacteroidota ratio, expansion of Proteobacteria and *Candida* spp., and depletion of short-chain fatty acid (SCFA)-producing bacteria—have been consistently associated with chronic inflammation, altered estrogen metabolism, and metabolic dysfunction. These associations support the concept of the gut–ovary axis as an important component of female reproductive health and suggest that the gut microbiome may become a valuable source of diagnostic and prognostic biomarkers.

However, current evidence remains limited by the predominance of cross-sectional studies, methodological heterogeneity, relatively small cohorts, and the lack of standardized protocols for sample collection, sequencing, and bioinformatic analyses. These limitations currently preclude causal inference and hinder direct translation of microbiome research into routine clinical practice.

Future research should therefore prioritize three key areas. First, large, well-powered longitudinal and multicenter studies are needed to validate reproducible microbiome signatures and determine their predictive value. Second, standardized methodological frameworks-including harmonized sampling procedures, sequencing strategies, and analytical pipelines-should be established to improve reproducibility and comparability across studies. Third, integration of metagenomics, metabolomics, and other multi-omics approaches with clinical phenotyping is essential to elucidate the mechanisms linking microbial dysbiosis to endocrine and immune pathways and to facilitate the development of personalized diagnostic and therapeutic strategies.

Although microbiome-based diagnostics are not yet ready for clinical implementation, improving early disease detection and risk stratification has the potential to enhance patient outcomes and may ultimately contribute to reducing the substantial healthcare and socioeconomic burden associated with chronic gynecological diseases.

## Figures and Tables

**Table 1:** Comparison of gut microbiota profiles in women with and without endometriosis at the taxonomic level. Data are presented as mean ± SD.

Taxon	Women with Endometriosis (Mean ± SD)	Control Group (Mean ± SD)	*p*-Value	Additional Information
Firmicutes	70.5 ± 15.2	68.3 ± 14.8	NS	Dominant phyla
Bacteroidetes	19.8 ± 10.4	21.6 ± 9.7	NS	Dominant phyla
Proteobacteria	7.2 ± 5.3	6.1 ± 4.8	*p* < 0.05	Increase reported in selected EMT cohorts
Actinobacteria	13 ± 8.1	4 ± 2.7	*p* < 0.05	Consistently reported increase in EMT
Fusobacteria	0.9 ± 1.2	0.4 ± 0.6	*p* < 0.05	Possible increase
Verrucomicrobia	1.2 ± 0.9	1.3 ± 1.0	NS	Ambiguous changes
Tenericutes	0 ± 0	2 ± 1.5	*p* < 0.05	Reported decrease in EMT
Spirochaetes	0.1 ± 0.2	0.1 ± 0.1	NS	Incidental reports
Firmicutes/Bacteroidetes	3.55	1.99	*p* < 0.05	Higher ratio reported in EMT; clinical relevance
Ascomycota	65 ± 10	63 ± 9	NS	Dominant in both groups
Basidiomycota	30 ± 8	32 ± 7	NS	Co-dominant
Mucoromycota/Chytridiomycota	5 ± 2	5 ± 2	NS	Rare

**Table 2:** Illustrative interpretation of Faith’s phylogenetic diversity (PD) reported in microbiome studies.

Faith’s PD	Interpretative Description	Clinical Significance	Potential Supportive Considerations
<10.0	Low phylogenetic diversity (dysbiosis-associated) [58,101]	Impoverished microbiome with reduced metabolic potential; increased susceptibility to inflammation and pathobiont expansion	Probiotics and lifestyle-based supportive strategies; further management based on comprehensive clinical assessment
10.0–18.0	Moderate phylogenetic diversity (intermediate, context-dependent) [77,82,100]	Limited functional flexibility; transitional state with increased susceptibility to environmental or host-related stressors	Probiotics, stress reduction, dietary changes with monitoring, as increased susceptibility to dysbiosis has been reported in women with subclinical endocrine or immunological symptoms
18.0–20.0	Eubiosis [84,102]	Functionally complex and resilient microbiome; association with mucosal protection, hormone metabolism, and immune homeostasis	Maintenance of a healthy diet, prebiotics

**Table 3:** Interpretation of the Shannon–Wiener index in the context of microbiota status, clinical significance, and recommendations.

Shannon–Wiener Index	Interpretative Description	Clinical Significance	Potential Supportive Considerations
<2.0	Dysbiosis (low α-diversity) [28,58,79,80,101,106]	Increased susceptibility to inflammation and hormonal dysregulation; possible association with impaired reproductive outcomes	Probiotics and lifestyle-based measures as supportive strategies; further management based on clinical context [107]
2.0–4.0	Intermediate diversity (context-dependent) [63,82,108]	Transitional or subclinical microbiota profile; variable functional impact	Probiotics, stress reduction, dietary modification with monitoring
>4.0	Eubiosis (higher diversity) [102,109,110]	Greater microbial stability and functional redundancy; association with metabolic and immune homeostasis	Maintenance of a balanced diet, prebiotics, avoidance of microbiota-disrupting factors

**Table 4:** Descriptive comparison of microbiome-derived indicators reported across studies of polycystic ovary syndrome/polyendocrine metabolic ovarian syndrome PCOS/PMOS, endometriosis, and uterine fibroids.

Microbiota Parameter	Healthy Women	PCOS/PMOS Women	Endometriosis Women	Fibroid Women	Clinical Significance	Metabolic Activity
Shannon–Wiener (α-diversity)	3.8–4.5 (approximate/synthesized ranges)	↓ 3.2–3.5	↓ 3.3–3.6 (or no difference in some cohorts; approx.)	↓ 3.4–3.7 (mild changes; inconsistent between studies)	High species richness (many taxa) and high uniformity (no taxon definitely dominates). Dominance of a few taxa, often opportunistic or pathogenic (e.g., *Gardnerella* sp., *Escherichia coli*, *Clostridium difficile*). Severely disturbed microbial balance—loss of specialized functional niches (e.g., estrogen degradation, colonization barrier, SCFA production)	A stable and balanced microbiome, resistant to colonization by pathogens. Strong immunoregulatory functionality: SCFA production (butyric acid, propionic acid), Treg induction, and mucosal barrier function. A state susceptible to disruption—the microbial barrier may be weakened. Increased risk of chronic inflammation (e.g., local inflammation in the endometrium), hormonal disruption (modification of estrogen metabolism by the microbiota), reactivation of infections (e.g., *Candida* sp., BV), and decreased fertility (especially when vaginal pathobionts are present) [72,79,104,106,107,110,111,112,118]
Faith’s PD	18.0–20.0	↓ 12–16	↓ 13.5–17	↓ 14–17.5	Reduced Faith’s PD is associated with a dominance of *Bacteroides* and *Escherichia*, and reduced numbers of short-chain fatty acid (SCFA)-producing bacteria, which correlates with insulin resistance and hyperandrogenism. Presence of phylogenetically similar taxa (e.g., a predominance of one large clade—Firmicutes or Bacteroidota). Possibly a decrease in functional specialization; less flexibility in metabolizing drugs or hormones. Dominance of related taxa (e.g., only *Lactobacillus iners* or Enterobacteriaceae). Lack of evolutionary diversity → poor functional capabilities (e.g., poor SCFA production, impaired estrogen metabolism)	Presence of taxa from different evolutionary lineages (e.g., Firmicutes, Actinobacteria, Bacteroidota, Proteobacteria). Functional richness—the ability to perform complementary metabolic reactions, resistance to stressors, and flexibility of the immune response. Presence of phylogenetically similar taxa (e.g., a predominance of one large clade—Firmicutes or Bacteroidota). Possibly a decrease in functional specialization, less flexibility in metabolizing drugs or hormones [63,72,82,92,101,110,111,118]
F/B ratio (Firmicutes/Bacteroidota)	1.5–2.0	↑ 2.5–3.5	~1.5–2.5 (often slightly increased)	↑ 2.0–2.8	Increased risk of insulin resistance and inflammation. Reduced fiber metabolism and increased endotoxins.	Increased risk of insulin resistance and inflammation. Reduced fiber metabolism; increased endotoxins [76,79,80,105,106,111,112,113,122]
Share of Proteobacteria in the microbiota profile	<2%	↑ 3–5%	↑ 3–7%	↑ 2–4%	Pathobiont growth; inflammation	LPS (endotoxin) production [76,79,101,116]
Share of *Akkermansia muciniphila* bacteria	0.5–5%	↓ <0.5%	↓ <1% (limited data)	↓ <1% (hypothetical; indirect evidence)	Weakened mucosal barrier. Decreased mucosal regeneration	Weakened mucosal barrier. Decreased mucosal regeneration [98,111,117]
*Bifidobacterium* spp.	5–10%	↓ 1–3%	↓ 2–4% (suggested)	~ normal or ↓	Weakening of immune protection. Decreased production of lactic acid and vitamin B	Weakening of immune protection. Decreased production of lactic acid and vitamin B [79,98,119]
*Candida* sp./Bacteria ratio	<1:1000	↑ >1:500	↑ >1:300	↑ >1:600	Predominance of fungi; mycodysbiosis; increased alcohol fermentation; cytotoxicity	Predominance of fungi; mycodysbiosis. Increased alcohol fermentation; cytotoxicity [58,74,116,120]
Total SCFAs (butyrate, acetate, propionate)	>15 mmol/kg	↓ <10 mmol/kg	↓ <12 mmol/kg	10–13 mmol/kg	Reduced barrier protection and energy metabolism	Reduced SCFA production; proinflammatory dysbiosis [101,107,115,121]
Acetate:Propionate:Butyrate (ratios)	60:20:20	70:15:15 or less	65:20:15	68:17:15	Change in the fermentation profile	Increased fermentation towards obesity [104,121]

All numerical ranges are approximate and synthesized from studies conducted in women with PCOS, endometriosis, or uterine fibroids. Values indicate trends rather than diagnostic cut-offs. Abbreviations: PCOS, polycystic ovary syndrome; PMOS, polyendocrine metabolic ovarian syndrome; PD, phylogenetic diversity; F/B, Firmicutes/Bacteroidota ratio; SCFA, short-chain fatty acid; LPS, lipopolysaccharide; BV, bacterial vaginosis. Symbols: ↑, increased; ↓, decreased.

**Table 5:** Technical problems and possible solutions in the analysis of bacteria and fungi as intestinal microbiota communities.

Problem Area	Technical Parameter	Impact on Bacterial Profiles	Impact on Fungal Profiles
Sampling	Fresh stool, frozen < 2 h (−80 °C) [58,63,79,106,111,112,130,131]	Preserves native community structure; prevents anaerobe degradation and F/B ratio shifts.	Maintains integrity of fungal DNA; essential for detecting low-abundance taxa (e.g., *Malassezia*, *Cladosporium*).
	Storage at room temperature without stabilizer [58,61,111,149]	Aerobic overgrowth; lysis of obligate anaerobes; artificial elevation of aerobes.	Proliferation of opportunistic fungi (e.g., *Candida albicans*); distortion of mycobiome signatures relevant to PCOS/endometriosis.
	DNA stabilizers (RNAlater, OMNIgene-GUT, DNA/RNA Shield) [58,79,134]	Stabilize bacterial proportions; may reduce efficiency of cell lysis for some taxa.	Maintain bacteria–fungi ratios; extraction may be hindered for fungi with rigid walls (requires bead-beating).
	Transport time & temperature [62,63,106,130,131,135,136]	Each hour at RT increases anaerobic degradation; long RT → community shifts. RT-stable kits mitigate loss but may interfere with downstream lysis.	Shift toward fast-growing aerobic fungi; destabilizes profiles in fertility/PCOS/endometriosis studies.
Genetic Analysis	Marker selection [58,61,111,112,139,140,150]	Different 16S regions (V1–V2, V3–V4, V4, V6–V8) provide non-equivalent taxonomic resolution; limits cross-study comparability.	Lack of standardized ITS region selection may contribute to inconsistent mycobiome results.
	Sequencing platform (Illumina vs. PacBio/Nanopore) [62,79,138,140,141,149]	Illumina: short, accurate reads; PacBio/Nanopore: long reads with better species resolution. Critical for differentiating *Lactobacillus* strains relevant to gynecological conditions	Long-read platforms improve species-level identification of rare fungi.

Abbreviations: F/B, Firmicutes/Bacteroidota ratio; DNA, deoxyribonucleic acid; RNA, ribonucleic acid; RT, room temperature; PCOS, polycystic ovary syndrome; PMOS, polyendocrine metabolic ovarian syndrome; 16S rRNA, 16S ribosomal RNA; ITS, internal transcribed spacer.

**Table 6:** Summary of the economic and social costs of endometriosis, PCOS, and uterine fibroids.

Aspect/Condition	Endometriosis [157,158,159,160,162]	PCOS/PMOS [163,164,167]	Uterine Fibroids [165,166,168]
Annual cost range (direct + indirect)	~€8768–$20,898 per patient per year in various countries (direct + productivity loss) [157,162]	Total economic cost of PCOS/PMOS in the US ~$7.9 billion annually [163,167]	In the US, total social costs increase to ~$41–42 billion annually in 2022 [165,168]
Direct medical costs	Cost of treatment and diagnostics: $1459–$20,239 per person per year [155,157,162]	PCOS/PMOS—diagnostic and initial treatment costs relatively low (~$139 million) [163]	Significant—surgical procedures, hospitalizations, therapies; increasing over time [166]
Indirect costs (productivity)	Productivity loss constitutes the major part of costs; in Australia ~84% of total losses (average ~$30,900 per person) [160,162]	PCOS/PMOS—costs related to diabetes and cardiovascular diseases account for ~48% of total costs [163]	Productivity loss after procedures and during work—an important component (~$6.4–30.2 billion) [166,168]
Costs of complications and comorbidities	Fertility problems, surgical procedures, long-term hormonal therapies [162]	PCOS/PMOS increases the risk of endometrial cancer—cost of care related to cancer alone is ~$467 million annually in the US [164]	Peripartum complications (cesarean sections, preterm births) increase disease-related costs
Social costs	Chronic pain and discomfort-reduced quality of life-problems with work/education [162]	Long-term metabolic disorders-psychological burden-reproductive problems and their consequences	Severe symptoms affecting daily life-post-procedural trauma-frequent medical visits
Global remarks	Endometriosis generates significant social costs, often underestimated due to diagnostic delays and the chronic nature of the disease	PCOS/PMOS is a chronic condition with numerous coexisting metabolic and reproductive disorders	Fibroids are one of the most common causes of gynecological procedures, increasing the burden on the healthcare system
Costs of infertility treatment	High—infertility affects approx. 30–50% of patients; cost of one IVF cycle: €3000–€6000 (EU)/$12,000–$15,000 (USA); multiple cycles often required	Moderate–high—PCOS/PMOS accounts for 70–80% of cases of anovulatory infertility; ovulation induction costs are lower, but IVF is common in treatment resistance; IVF cost as above; significant long-term costs of hormonal therapy	Variable-high-infertility in 5–10% of women; increased costs due to surgical procedures (myomectomy) + IVF; cost of myomectomy before IVF $5000–$15,000, followed by ART costs

Abbreviations: PCOS, polycystic ovary syndrome; PMOS, polyendocrine metabolic ovarian syndrome; IVF, in vitro fertilization; EU, European Union; USA, United States of America; ART, assisted reproductive technology.

## Data Availability

The data used in this review were extracted from publicly available published studies and are summarized within the article. No new primary or participant-level data were generated, and no analytic code was used.

## Supplementary Materials

The following supporting information can be downloaded at: https://www.mdpi.com/article/10.3390/jcm15176705/s1. File S1: PRISMA checklist [172].
